# Robust Single-Sample Face Recognition by Sparsity-Driven Sub-Dictionary Learning Using Deep Features †

**DOI:** 10.3390/s19010146

**Published:** 2019-01-03

**Authors:** Vittorio Cuculo, Alessandro D’Amelio, Giuliano Grossi, Raffaella Lanzarotti, Jianyi Lin

**Affiliations:** 1 Dipartimento di Informatica, Università degli Studi di Milano, via Celoria 18, 20133 Milano, Italy; vittorio.cuculo@unimi.it (V.C.); alessandro.damelio@unimi.it (A.D.); giuliano.grossi@unimi.it (G.G.); 2 Department of Mathematics, Khalifa University of Science and Technology, Al Saada Street, PO Box 127788, Abu Dhabi, UAE; jianyi.lin@ku.ac.ae

**Keywords:** face recognition, single sample per person, dictionary learning, optimal directions (MOD), Deep Convolutional Neural Network (DCNN) features, sparse recovery

## Abstract

Face recognition using a single reference image per subject is challenging, above all when referring to a large gallery of subjects. Furthermore, the problem hardness seriously increases when the images are acquired in unconstrained conditions. In this paper we address the challenging Single Sample Per Person (SSPP) problem considering large datasets of images acquired in the wild, thus possibly featuring illumination, pose, face expression, partial occlusions, and low-resolution hurdles. The proposed technique alternates a sparse dictionary learning technique based on the method of optimal direction and the iterative $\ell_{0}$-norm minimization algorithm called *k*-LiMapS. It works on robust deep-learned features, provided that the image variability is extended by standard augmentation techniques. Experiments show the effectiveness of our method against the hardness introduced above: first, we report extensive experiments on the unconstrained LFW dataset when referring to large galleries up to 1680 subjects; second, we present experiments on very low-resolution test images up to 8×8 pixels; third, tests on the AR dataset are analyzed against specific disguises such as partial occlusions, facial expressions, and illumination problems. In all the three scenarios our method outperforms the state-of-the-art approaches adopting similar configurations.

## 1. Introduction

Faces convey a plethora of information, such as expression, gender, age, ethnic origin, and identity. Indeed these factors co-exist and the ability to recognize each of them is strictly correlated with the capability to isolate one from the others. This task is made even harder by the different characteristics of each factor. For example, expressions are transient, change fast and significantly, while aging is permanent, and affect the face appearance gradually. Performing a person identification requires to disregard these changes and recover the immutable characteristic of the identity. The problem is made further complex by the face appearance variations caused by head pose changes and by possible external factors, such as variation of illumination or presence of partial occlusions. Despite this complex scenario, humans are extremely gifted in solving this task, while for automatic systems it is still a challenging problem, being further complicated by possible image corruption (noisy or blurring) due to either the employment of low-cost sensors or to large distances between the subjects and the acquisition cameras. Recently, Face Recognition (FR) has seen a breakthrough mainly thanks to the introduction of deep neural networks [1,2], thus allowing its adoption in plentiful applications [3]. Even though, there are still several open problems [4] deserving further investigation. The main challenges concern the long-standing difficulties of dealing with images acquired in unconstrained conditions [5], implying the necessity to deal with several illumination conditions, head poses, facial expressions, possible partial occlusions, and possible low image quality [6]. Furthermore, the matter gets more difficult by the double hardness of accomplishing the recognition task dealing with large-scale databases [7], and having only a few images per subject available for the gallery/train construction, facing the so called *Small Sample Size* (SSS) problem, or even the extreme case when only one image is available: the *Single Sample Per Person* (SSPP) problem [4,8]. Such challenge is of leading interest in application such as e-passport control, law enforcement, surveillance, human-computer interaction, to name just a few. Furthermore, even harder challenges are the scenarios where these problems co-exist, requiring the conception of powerful and robust methods for SSPP, able to deal with images possibly corrupted. Recently, a large investigation effort has been put on this research field, achieving promising but not yet satisfactory results [4]. In Section 2 we recall and organize the most recent contributions to draw an up-to-date picture of this domain.

In this paper we propose a SSPP method robust to low-quality images and disguised face images (Section 3). It extends a preliminary study [9] where the SSPP problem was faced by combining deep-learned features with the sparse representation paradigm. Specifically, the VGG-face net [10] was adopted to achieve highly discriminative features, and the *k*-LiMapS algorithm [11,12] to accomplish the goal of deriving a concise description of a test image on a collection of feature dictionaries. Consequently, the FR problem was recast as mere counting of presence of labeled atoms over all codings, i.e., using majority voting. Here two main novelties are introduced:

- *Face augmentation step*: we enrich the character of the discriminative features by producing a very large collection of augmented images (considering several scales, crops, displacements and filtering). This way, besides facing the hurdle of availing of a SSPP for the gallery construction, we make the system robust to *partial occlusions* (collecting face sub-portions dual to the occlusions), *multi-poses* (parts of the faces are less sensitive to pose than the whole face), and *low resolution* (characterizing even very low-quality image versions).
- *Sparse sub-dictionary learning step*: given the huge quantity of data produced with the face augmentation step, it is essential to derive a *space* suitable for the classification, together with a succinct and effective *model* underlying the data. The feature space is obtained employing deep features coupled with the linear discriminant analysis, while the concise model is derived adopting the method of optimal directions (MOD) [13], which has proved to be very efficient for low-dimensional input data. The benefits of this approach is that, contrarily to generic learning algorithms [14], the label consistency between dictionary atoms and training data is maintained, allowing the direct application of the classification stage based on majority voting (a demo code is available on the website: https://github.com/phuselab/SSLD-face_recognition).

Essentially, the core idea in our Sparsity-driven Sub-dictionary Learning using Deep features (SSLD) technique is to work out a large number of face augmentation, characterize them with very discriminative deep features, derive a succinct sub-dictionary for each subject through *k*-LiMapS sparse optimizer, and deduce the identity of probe images by combining multiple classifications by the majority voting. This pipeline allows to deal with SSPP problem coupled with several further nuisances, while keeping the system very efficient, and thus suitable for real-world applications. A further advantage of this approach is that it does not require any additional generic dataset for learning, which collection would pose further issues to avoid overfitting while promoting a good generalization capability. The effectiveness of the method is proven in Section 4: the LFW dataset is adopted to evaluate the robustness against uncontrolled conditions with large gallery sets, the AR database is tested to analyze the method behaviour against natural occlusions (people wearing sunglasses or scarf), illuminations and face expressions. Finally, we investigate the method robustness against low-resolution probe images by degrading the LFW images. In Section 5 we highlight the key points of our work and draw potential future directions in this domain.

## 2. Related Works

The methods dealing with the SSPP problem can be grouped in three categories [4]: (i) learning methods, which characterize possible face changes referring to a distinct and rich face image set so to attribute them to the available scarce labeled data; (ii) generative methods, that devise new synthetic images starting from the available reference ones, so enriching the gallery set, and (iii) local methods, which achieve a higher discerning power thanks to face local characterizations.

### (i) Learning Methods

These approaches aim at recovering the face without variants (or at least to attenuate them), and extracting robust features for image representation so to reduce the intra-class variance. The hypothesis common to these methods is to avail of a generic training set, suitable to characterize the nuisance variations expected in the test set.

In [15] Deng et al. proposed a method aiming at mapping gallery images to equally distant positions in an embedding space, disregarding the data structure, and simultaneously reducing to zero vectors the intra-class facial differences, so to augment the method robustness. These goals are attained adopting a least square regression technique (LRA) formulated as the generalized inverse of the training data matrix, incrementally computed adopting the well-known Greville algorithm. This approach achieves the same performance as the batch LRA, while allowing efficient update when adding new subjects in the gallery. The authors extensively test their method on four datasets acquired in controlled conditions (Ext.Yale B, CMU-PIE, AR, and FERET), showing the effectiveness of the generic learning. In Section 4 we report the results obtained by LRA on the AR dataset, comparing them with our method. In the same vein, Hu et al. [16] adopted a discriminative transfer learning (DTL) approach for SSPP. Given a large generic dataset, containing multiple images per subject, and the gallery set, including a single sample per subject, DTL learns a model that maximizes the intra-class variation and minimizes the inter-class one on the generic dataset, simultaneously minimizing the distribution difference between the two datasets in a joint latent subspace via manifold alignment strategy. A sparsity regularizer is also added to increase the generalization capability. Experiments conducted on the FERET and CAS-PEAL-R1 datasets show the validity of the method, while the tests on the LFW highlight the limits of the method (cfr. Section 4.1). In [17] the authors proposed a fully automatic method for FR in uncontrolled environments. In order to cope with pose variations, cause of major problems in real-world FR systems, their method first pre-process the images with a pose normalization technique based on piece-wise affine warping transformation that can work out well both in-plane and out-of-plane pose changes. The warping is applied on a triangular mesh determined by an enhanced active appearance model (AAM) where landmark location initialization is performed with a landmark mixture strategy. The algorithm is then evaluated against the FERET (b-series) and the CMU-PIE databases on galleries containing respectively 200 and 68 single samples per subject in normal conditions (neutral expression, frontal pose, …), outperforming the state of the art. Also the experiments conducted on the LFW dataset achieve high accuracy, while referring to multi-sample gallery and so not framed in the SSPP problem. Another effective approach is presented in [18] where the reduction of the intra-class variability is achieved training a supervised auto-encoder to map all the faces with their peculiar appearance (e.g., illumination, pose, expression) onto the canonical face of the person, normalizing them. The authors propose a Stacked Supervised Auto-Encoders (SSAE) where the activation function of the encoder is applied to both normalized and corrupted data, and their outputs are the input to the next layer, determining a deep architecture. FR experiments are conducted on the Ext.Yale B, CMU-PIE, and AR datasets, while LFW is adopted for the face verification problem. Performances are not outstanding, while the approach is original and well established. Chu et al. [6] face the very challenging low-resolution FR with SSPP problem. The authors proposed a cluster-based regularized simultaneous discriminant analysis (C-RSDA), aiming at regularizing both the inter-class and intra-class scatter matrices. The method conceives the employment of two scatter matrices, a cluster-based and a class-based, to learn a mapping suitable to project both high-resolution (HR) and low-resolution (LR) images into a common space, suitable for performing the recognition step. Extensive experiments have been conducted highlighting the effectiveness of the method on controlled databases such as the FERET, and the limits on unconstrained images (cfr. Section 4.2 for comparisons on LFW the dataset).

### (ii) Generative Methods

Methods in this category aim at overcoming the lack of training samples by expanding virtually the gallery at hand. Specifically, intra-class variations are first learned from a disjoint set of images and then applied to the gallery single samples to obtain intra-class variations, thus expanding their expressiveness.

In [19], given single training images and exploiting a sufficiently rich bootstrap set, the method produces the corresponding 3D face rendering, being potentially able to synthesize images with any pose or illumination variations. This is achieved coupling a multi-depth 3D generic elastic model with the quotient image technique, aiming at synthesizing virtual faces with a desired illumination and expression, given a frontal image. A pose-aware metric learning method then transforms each model into a single point in a suitable metric space. The single image classification is achieved by first estimating the pose of the face and then applying the pose-specific metric classification. Experiments on the Multi-PIE dataset show the effectiveness of the method, that outperforms by 10% recently proposed deep learning methods. Gao et al. [20] introduced a method to deal with both the SSPP and the possible presence of nuisance variables (both linear and non-linear). The technique, called S${}^{3}$RC, is a sparsity-based classification method grounded on a couple of dictionaries: the gallery and the variation one. The first aims at characterizing the face identity, while the second is conceived to capture possible variations. The residual error of a sample is modeled as a Gaussian Mixture Model noise, whose parameters (centroid and covariance matrix) are estimated in maximum likelihood sense by an EM algorithm initialized with the empirical distribution as class prior. Hence, the estimated centroids form the gallery dictionary, while the variation dictionary is obtained from single samples or from standard within-class centering of labeled samples. The so-constructed minimum $\ell_{2}$ residual classifier is experimented on the AR and the LFW databases, while only in the latter case referring to the SSPP problem (cfr. Table 1). In [21] another method based on sparse coding is proposed. Here, Yu et al. establish their method on a dictionary learned from a generic unlabeled dictionary, aiming at modeling possible occlusions. Given a disguised test image, the method is able to localize pixels affected by occlusions by means of a multi-scale error measurement technique. The method setups the training on the images corresponding to 20 subjects of the AR dataset, while tests are performed on other 80 subjects of the same dataset. Despite the performances are very good, we claim the learning on a subset of a so specific dataset does not allow generalization of the performance to more realistic and unconstrained scenarios. In [22] a collaborative representation and probabilistic graph model is proposed. Two dictionaries are constructed: the gallery dictionary, based on the SSPP training images, and an adaptive probabilistic label dictionary, exploiting a distinct unlabeled image set, congruous for characterizing possible variants in the test set. To handle the testing phase, a reconstruction-based classifier is adopted and tested on a subset of 70 subjects of the AR dataset, achieving good but not outstanding recognition rates (90.65% on images without occlusions, 85.65% and 72.37% on images with occlusions in the first and second session respectively).

### (iii) Local Methods

Local methods have their strength in characterizing patches of the faces, under the hypothesis that possible nuisance (e.g., partial occlusions, expression chances, and illumination variations) affect only a part of the face. Collaborative representation should thus exclude outliers from the decision and reach a robust classification.

For example, in [23] an iterative $\ell_{2}$-regularized CRC method is proposed, grounding on local structure of decomposed sample images. More specifically, sub-dictionaries built on local patches are structured in a complete dictionary and referred during testing phase: probe images are first decomposed in patches as the training ones, then each image portion undergoes an iterative process where the CRC method is alternated with a pruning phase aiming at promoting sparsity. The process stops when a satisfactory majority voting is achieved. The experiments on the AR dataset indicate an improvement compared to state-of-the-art alternatives (cfr. Table 3), while low performances are attained on the LFW dataset (cfr. Table 1). Another joint collaborative representation model is proposed in [24], effectively fusing the deep-feature representations corresponding to different image portions. Among the others, experiments on the AR and LFW datasets are conducted (cfr. Table 1). A block-based partition of face images is adopted also in [25] and [26]. The first paper proposes to characterize the blocks applying a kernel principal component analysis network (KPCANet), while the second refers to a variation dictionary learned from external data. Both methods have been tested on the LFW dataset as reported in Table 1. Finally, we recall the method proposed by Pei et al. [27] that characterizes each block extracting the LBP features and attains the FR adopting the decision tree technique. Experiments on 100 subjects of the AR dataset produced on average 83% of recognition rate.

## 3. Method

**Figure 1:**
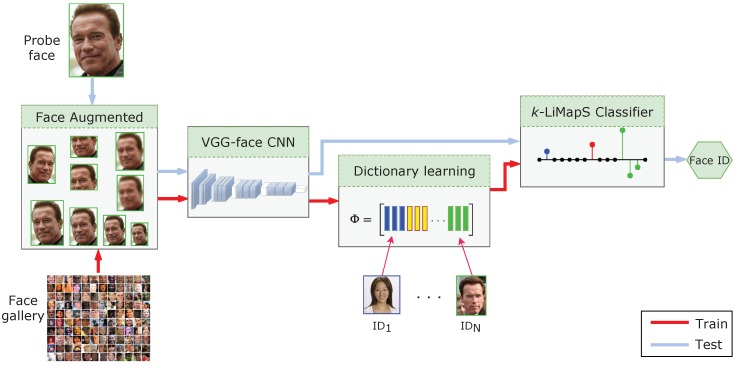
Classification process diagram. First stage: gallery and probe image augmentation. Second stage: deep-feature extraction via VGG-face net. Third stage: sparsity-driven sub-dictionary learning. Fourth stage: identity characterization by *k*-LiMapS and face identity finding.

In this section we describe a sparse-driven sub-dictionary learning technique, applied on highly discriminative characterizations obtained by deep CNN (SSLD). In Figure 1 the classification process is sketched: we start applying simple transformations (such as scale reduction, cropping and flipping) to the unique sample available in the gallery.

Next, a highly discriminative characterization of the augmented image set is obtained applying the VGG-face net [10], and subsequently the linear discriminant analysis (LDA) that reduces the feature dimensionality. This paves the way to the dictionary building, applying the MOD as sparse dictionary learning technique. Lastly, adopting the learned dictionary, we leverage on the *k*-LiMapS algorithm $\ell_{0}$-norm minimizer [12] to derive a sparse coding of the test images, and solve the FR task. We describe the details of these stages in the remaining subsections hereafter.

### 3.1. Deep Features on Geometrical Transformations

SSPP can be tackled either using the single-sample reference images available [28], or by enriching the gallery. Our approach, being based on sparse classification, requires populating the dictionary with multi-sample per subject, so to derive a low-rank subspace characterizing each subject in the training gallery. To attain the augmented images, as recalled in Section 2 and well studied in [29], one could adopt either learning methods, based on the generative adversarial networks, or 3D model-based methods, or traditional affine transforms. The first two ways constitute challenging open research problems *per se*. Besides their intrinsic complexity, a further not negligible aspect is that they both require a large generic dataset to learn virtual samples. On the contrary, the third method works directly on the available data, applying to them simple transformations such as flipping, scaling and multi-cropping, thus enriching the image characterization. Of course, this enrichment covers only partially the possible face poses and more in general the possible nuisances that could affect the face images. This challenge motivates the adoption of the subsequent steps, aiming at generalizing from the augmented data with a dictionary learning step, and dealing with unavoidable discrepancy between the dictionary and probe image representations with the robust *k*-LiMapS sparsity promotion.

Specifically, each face image *I* is first normalized [30], and then a pool of *d* transformations is applied on *I* providing the set of new augmented images $A_{I}=\left\{I_{1},\cdots ,I_{d}\right\}$. To be successful in classification, it turns out to be useful to project each augmented face image $I_{j}\in A_{I}$ onto a proper feature space capturing relevant visual content of the image itself. In the vein of Gao et al. [20], we derive a highly discriminative feature characterization resorting to deep CNN, adopting the VGG-face net presented in [10]. It is a public deep convolutional neural network (DCNN) conceived for the FR task, thus suitable to extract complex and even subtle face characterizations. Specifically, we refer to the output of the last full connected layer: for each augmented image $I_{j}^{i}$ of subject *i*, with j∈D={1,⋯,d}, we work out the characterization $\varphi_{j}^{i}=\mathrm{VGG-face}\left(I_{j}^{i}\right)$, which is a *p*-dimensional sparse vector (p=4096). The obtained features are hence arranged in the matrix $F_{i}=\left[\varphi_{1}^{i}|\cdots |\varphi_{d}^{i}\right].$

### 3.2. Feature Projection into LDA Space

Let C={1,⋯,q} be a set of subjects, each with a unique reference image, and suppose we are given a probe image of the subject s∈C. Before applying some learning process, it is very common to transform the data into some suitable space where the power of distinguishing among sample vectors of different subjects is improved. Such discriminative capability can be pursued by applying Fisher’s LDA [31], a method largely used in pattern recognition and machine learning. LDA works out a succinct and highly discriminative characterization, projecting the available data into a space with strongly reduced dimensions, aiming at having the best-possible class separability. We outline the application of LDA to our problem according to Fisher’s original approach [32] where no normal probability distribution assumption is posed at all, but rather a linear combination of explanatory features is sought to maximize the Fisher’s ratio. This is an empirical measure of between-class separability over pooled within-class homogeneity in the transformed feature space.

To apply this technique in our setting, given a matrix of features $F=[F_{1}|F_{2}|\cdots |F_{q}]$ accounting for all the *q* subjects/classes in C, let

$$\mu =\frac{1}{qd}\sum_{i=1}^{q}\sum_{j=1}^{d}\varphi_{j}^{i}\;\mathrm{and}\;\mu_{i}=\frac{1}{d}\sum_{j=1}^{d}\varphi_{j}^{i}$$

be the global mean and the mean of class *i* respectively, and let

$$S_{W}=\sum_{i=1}^{q}\sum_{j=1}^{d}\left(\varphi_{j}^{i}-\mu_{i}\right){\left(\varphi_{j}^{i}-\mu_{i}\right)}^{T}\;\mathrm{and}\;S_{B}=d\sum_{i=1}^{q}\left(\mu_{i}-\mu \right){\left(\mu_{i}-\mu \right)}^{T}$$

be the within-class scatter matrix and the between-class scatter matrix, respectively. The Fisher’s discriminant analysis determines a weight matrix $W\in R^{(q-1)\times p}$ that projects all high-dimensional data $\varphi_{j}\in F$ in the reduced feature space $R^{q-1}$ aiming at maximizing class separability of the projected feature vectors. *W* is obtained by optimizing the functional

$$J\left(W\right)=\frac{|W^{T}S_{B}W|}{|W^{T}S_{W}W|},$$

and is proven to be an optimal for the generalized Rayleigh quotient criterion. From numerical computation viewpoint, this is carried out by solving generalized eigenvalue problems [33]:

$$S_{B}w=\lambda S_{W}w$$

and since $\mathrm{rank}(S_{B})\leq q-1$, in the non-degenerate cases we obtain *W* stacking the q−1 generalized eigenvectors *w* regarded as row vectors.

The LDA technique is adopted to transform feature vectors for both gallery and probe images, which will be referred in the subsequent learning and classification steps, as detailed in the next section. The new features, lying in the LDA space and denoted with the superscript LDA, should be computed as follows:

(1) $$\begin{array}{cc} F^{\mathrm{LDA}} & =WF \end{array}$$

(2) $$\begin{array}{cc} \psi_{j}^{\mathrm{LDA},i} & =W\;\psi_{j}^{i},\;\mathrm{for}\;\mathrm{all}\;j=1,\cdots ,d \end{array}$$

where last equation holds for the features of the probe subject *i*. For the sake of readability, we relieve the notational burden by dropping the LDA superscript henceforth.

We remark that, as classical consequence of applying the LDA, the transformed feature vectors have reduced dimensionality compared to the high dimensionality of VGG-face net features.

### 3.3. Sparse Sub-Dictionary Learning and Representation

Before describing the learning process applied to LDA features, here we briefly introduce the linear sparsity model.

#### 3.3.1. Sparse Representation

The general framework of sparse representation consists in exploiting the linear combination of some prototype samples or atoms to represent a probe sample. Given a collection of known atoms $\phi_{i},\cdots ,\phi_{m}$ such that $\phi_{i}\in R^{n}$ for all i=1,⋯,m, if m<n the matrix $\Phi =[\phi_{1},\cdots ,\phi_{m}]$ where atoms are arranged as columns is called over-complete dictionary. Let us consider a linear system of equations Φx=s for a given probe sample $s\in R^{m}$. From the viewpoint of linear algebra the latter is an underdetermined linear system and then ill-posed because it does not have a unique solution. To overcome this multiplicity, it is reasonable to impose an appropriate regularization constraint as, for instance, to require that the obtained representation solution should be sparse. To find a *sparse* decomposition of the sample *s*, that is a solution *x* with very few nonzero terms, we can solve the following combinatorial optimization problem

(*P*_0_) $$\underset{\alpha \in R^{m}}{\mathrm{argmin}}{∥x∥}_{0}\;\mathrm{subject}\;\mathrm{to}\;\Phi x=y.$$

where ${∥x∥}_{0}=\left|\left\{j:x_{j}\neq 0\right\}\right|$ denotes the $\ell_{0}$-norm (strictly speaking the $\ell_{0}$-norm is not actually a norm, it is the function counting the number of nonzero elements in a vector *x*, satisfying the norm axioms but the absolute homogeneity.). This approach to sparsity is often referred as $\ell_{0}$-minimization. Note that problem (*P*_0_) is combinatorial in nature and hence NP-hard [34]. Moreover, when at most *k* atoms (with k<n) are sufficient to represent the sample *s*, the previous problem can be recasted in the following combinatorial problem of finding *x*:

$$y={\Phi x\;\mathrm{subject}\;\mathrm{to}\;∥x∥}_{0}\leq k,$$

which is often referred to as *k-sparse approximation problem*. In this case, the feasible set is the union of lower dimensional subspaces generated by canonical basis vectors.

Since data in real applications often contains noise, the model appearing in the previous equation is sometime unrealistic. Thus, it is reasonable to revise such exact model introducing a small possible noise by defining the problem Φα=s+ε, where $\varepsilon \in R^{n}$ refers to a representation noise which is in general a bounded quantity, i.e., ${∥\varepsilon ∥}_{2}\leq \sigma$. Therefore, under the noisy model assumption, the problem (*P*_0_) can be approximately solved by addressing the combinatorial problem

(*P_a_*) $$\underset{\alpha \in R^{m}}{\mathrm{argmin}}{∥\Phi x-s∥}_{2}^{2}\;\mathrm{subject}\;\mathrm{to}\;{∥x∥}_{0}\leq k.$$

We tackle the sparse representation problem (*P_a_*) by resorting to the *k*-LiMapS [12] regularization method. Essentially, it relies on a fixed-point iteration scheme which combines non-convex Lipschitzian-type mappings with canonical orthogonal projectors. The first are aimed at uniformly enhancing the sparseness level by shrinking effects, while the latter to project back into the feasible space of solutions. A motivated reason to use *k*-LiMapS is that we have already demonstrated in past works its ability to find low-rank approximate solutions in tasks such as biomedical signal compression [35] and FR problems with very few training samples [36,37], and FR in presence of partial occlusions [38]. Here we show how to apply it to the SSPP problem which is one of the most challenging task in the realm of face analysis, as highlighted at the beginning of this paper.

#### 3.3.2. Sparse Dictionary Learning

In particular, in this work we make use of the sparse representation paradigm for deriving discriminative class-specific sub-dictionaries able to capture the sparsity pattern within the image classification context designed above. In order to minimize the reconstruction error among all the classes, we combine the well-known MOD with the sparsity representation on the structured dictionary provided by *k*-LiMapS.

More formally, given a collection of *d* features $F_{i}$ in LDA space for each subject *i*, we want to learn a corresponding dictionary $\Phi_{i}=\left[\varphi_{1},\cdots ,\varphi_{k}\right]$ of very few atoms, i.e., for k≪d. We define the structured dictionary as a matrix collecting all sub-dictionaries, one for each subject in the gallery, that is a frame of kq atoms of the form $\Phi =[\Phi_{1}|\cdots |\Phi_{q}]$. Following the same scheme, we define the matrix $X=[X_{i}|\cdots |X_{q}]$ by arranging in a unique row the submatrices $X_{i}\in R^{kq\times d}$ which encode the features $F_{i}$ using the dictionary Φ.

The rationale underneath this design is that the subject-specific dictionaries $\Phi_{i}$ are learned to well represent the face characteristics in the transformed LDA space through the sparse encoding submatrices $X_{i}$, that trigger only the atoms belonging to each specific subject respectively. This leads in the identity classification stage to a representation of the probe image that involves the dictionary of the true subject only.

Following this rationale we formulate the *sparse dictionary learning problem*

(3) $$\begin{array}{cc} (\hat{\Phi },\hat{X})= & \underset{\Phi \in R^{(q-1)\times kq},X\in R^{kq\times dq}}{\mathrm{argmin}}{∥F-\Phi X∥}_{F}^{2}\\ & \mathrm{subject}\;\mathrm{to}\;\;\;∥\varphi_{j}{∥}_{2}=1,\;j=1,\ldots ,kq\\ & \;\;\;∥x_{j}{∥}_{0}\leq k,\;j=1,\ldots ,dq \end{array}$$

where $\varphi_{j}$ and $x_{j}$ represent the *j*-th column of Φ and *X*, respectively.

#### 3.3.3. Computational Scheme

The search for an optimal solution of problem (3) is a well-known difficult task due both to the combinatorial nature of the problem and to the strong non-convexity given by the constraint on the $\ell_{0}$-norm. We tackle this problem adopting the well-established alternating optimization scheme [39], which consists in repeatedly executing the two steps:

1. *Sparse coding*: solve problem (3) for *X* only, fixing the dictionary Φ;
2. *Dictionary update*: solve problem (3) for Φ only, fixing X.

Of course, an effective algorithm prescribes also an initial feasible solution $\Phi^{0}$ used for starting the iterations, for example selecting a subset of *k* feature columns from *F* for every subject. The scheme is iterated until a stopping criterion is reached, that could be for instance the residual error below an acceptable threshold or a suitable predefined number of iterations.

To calculate the solution for the Step 1, that is the atom representation coefficients, many optimization heuristics could be used, each one being characterized by a different type of norm minimization incorporating the sparsity constraints [40]. In this work we concern with the strict $\ell_{0}$-norm minimization that is undertaken by the above-mentioned iterative algorithm *k*-LiMapS working on feature space both for dictionary and probe images.

The technique we utilize for carrying out Step 2 is the classical MOD [13]. This method consists, firstly, in locally minimizing the convex objective function of problem (3) regarding each sub-dictionary $\Phi_{i}$, without regard to the constraints. This minimization is quite straight-forward, since it gives rise to a least squares problem which projects the solution onto the convex set of admissible solutions, i.e.,

$${\hat{\Phi }}_{i}=F_{i}X_{i}^{†}$$

where $X_{i}^{†}$ denotes the Moore-Penrose pseudoinverse matrix [33] of $X_{i}$. Secondly, it rescales each atom $\varphi_{j}$ to fit the unit $\ell_{2}$-norm constraint, i.e., every atom is projected on the unit (q−2)-sphere $S^{q-2}$ centered at the origin of the LDA space. With such rescaling, the dictionary turns out to be no more optimal regarding the objective function and for the given matrix *X*, but in the subsequent Step 1 iteration of the alternating scheme, the sparsity encoding matrix *X* is re-encoded for the pursuit of reducing the residual error. The learning process described above is sketched in Algorithm 1.

**Algorithm 1:** SSLD: Learning Step

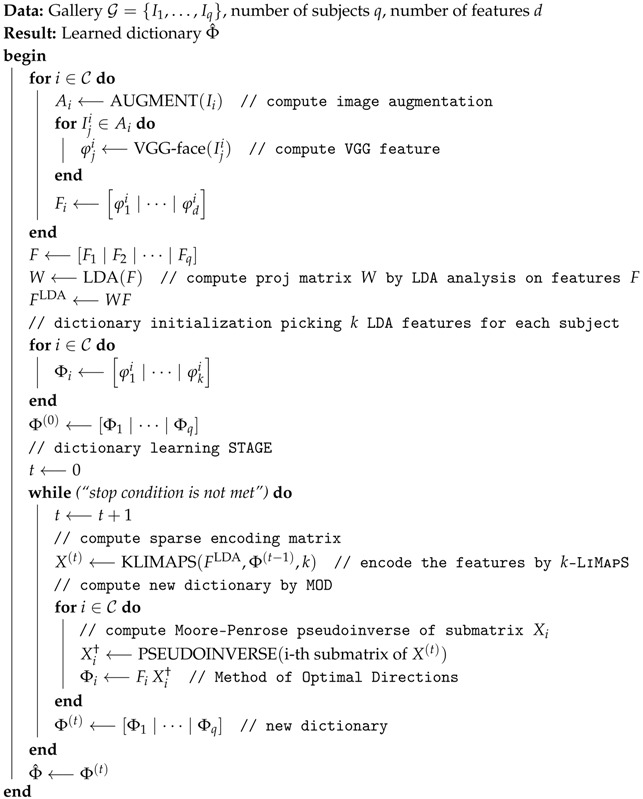



### 3.4. Identity Recovery via *k*-LiMapS Sparsity Promotion

As motivated in the previous section, the problem of recognizing a probe image against a close set of subjects, can be tackled seeking the *k*-sparse solution of a linear system characterizing each reference subject with *k* atoms. This consideration paves the way for the conception of our classification method, procedurally described in the process below:

1. according to (1), for the whole pool *D* of features *F* build the LDA projected features $F^{\mathrm{LDA}}\in R^{(q-1)\times dq}$, where *q* is the number of subjects in the gallery,
2. for a test face image $I^{i}$ of identity i∈C, work out the LDA projections $\psi_{j}^{\mathrm{LDA},i}$ from the feature vectors $\psi_{j}^{i}$ for every j∈D (Equation (2)),
3. for each feature, i.e., for all j∈D, solve the problem (*P_a_*) consisting of finding the *k*-sparse solution ${\hat{\alpha }}_{j}$ satisfying
  (4) $${\hat{\alpha }}_{j}=\underset{\alpha \in R^{kq}}{\mathrm{argmin}}∥{\hat{\Phi }}^{\mathrm{LDA}}\alpha -\psi_{j}^{\mathrm{LDA},i}{∥\;\mathrm{subject}\;\mathrm{to}\;∥\alpha ∥}_{0}\leq k$$
  where ${\hat{\Phi }}^{\mathrm{LDA}}$ results from the dictionary learning problem (3) applied to $F^{\mathrm{LDA}}$ in the LDA space.

This approach exploits the covariance among atoms belonging to different sub-dictionaries: setting the sparsity level of the linear system solution at the same value *k* of the subject sub-dictionary dimension, aims at activating all and only the atoms in ${\hat{\Phi }}^{\mathrm{LDA}}$ corresponding to the identity *i* of the test image at hand, as shown in the following

$${\hat{\Phi }}^{\mathrm{LDA}}=[\varphi_{1}|\cdots |\varphi_{k}|\cdots \underset{\mathrm{subject}\;i}{\underset{︸}{|\varphi_{(i-1)k+1}|\cdots |\varphi_{ik}|}}\cdots |\varphi_{(q-1)k+1}|\cdots |\varphi_{qk}].$$

In other words, the probe image features $\left[\psi_{1}^{\mathrm{LDA},i}|,\cdots ,|\psi_{d}^{\mathrm{LDA},i}\right]$ and those atoms highlighted above should have a high mutual coherence [41], and therefore the latter atoms pertaining to the subject *i* are largely preferable to the remaining ones for the probe image representation.

Notice that generally, when referring to a sparse solution, the matter is to minimize the residual measures (e.g., least squares minimization) over the weighted linear combination of atoms. Differently, here we leverage only on the support (for a given of vector α, the support $\mathrm{supp}\left(\alpha \right)=\left\{i:\alpha_{i}\neq 0\right\}$ is the index pool of nonzero entries of α.) of the sparse solution of Equation (4), claiming that this brings to a higher recognition rate even in presence of strong nuisances. Specifically, given the set $A=\left\{{\hat{\alpha }}_{1},\cdots ,{\hat{\alpha }}_{d}\right\}$ of *d* sparse solutions associated with the probe image $I^{i}$, we consider the support of each of them, and define a rather natural voting approach for the identity recognition:

1. Let L:{1,⋯,kq}→C be the function that maps the column-index *t* of $\Phi^{\mathrm{LDA}}$ to the subject in C corresponding to the atom $\varphi_{t}$,
2. define
  $$V_{j}=\left\{L\left(t\right)\in C:t\in \mathrm{supp}\left({\hat{\alpha }}_{j}\right)\right\}$$
  as the set of identity votes casted by the *j*-th feature, j=1,…,d,
3. collect the votes together in the multi-set $V={⋃}_{j=1,\cdots ,d}V_{j}$ and, if the mode of *V* is unique, determine the subject identity consequently
4. otherwise, apply the least squares residual criterion between the probe features of every winner and the linear combination of their respective dictionary atoms, so as to achieve a subject ranking.

The identity recovery process described above is sketched in Algorithm 2. 

**Algorithm 2:** SSLD: Identity Recovery

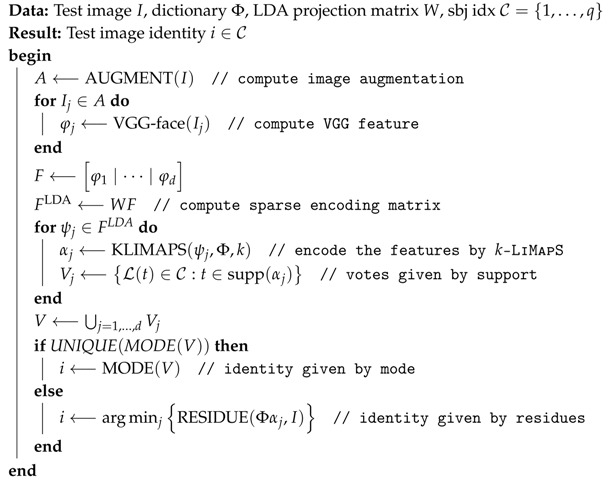



## 4. Experimental Results

In this section, we evaluate the effectiveness of the proposed SSLD method facing the SSPP problem, together with the three main challenges recalled in the introduction: large gallery cardinality, LR (low-resolution) probe images, and disguised test images. For the first two experiments we adopt to the LFW funneled dataset [42], containing more than 13,000 images of 5749 different people acquired in uncontrolled conditions. The pose, illumination, and expression variations, together with the possible presence of partial occlusions and disguised faces make SSPP problem extremely challenging. The third experiment is performed adopting the AR [43], that contains more than 4000 images of 126 subjects acquired in two sessions, each session containing 13 images. The images in the AR database are characterized according to the illumination changes, the face expression and facial occlusions, allowing to analyze the system behaviour in each scenario separately. Notice that, while the LFW funneled images are already centered and normalized, the AR faces require a normalization step that we accomplish using the landmark detector presented in [30].

**Figure 2:**
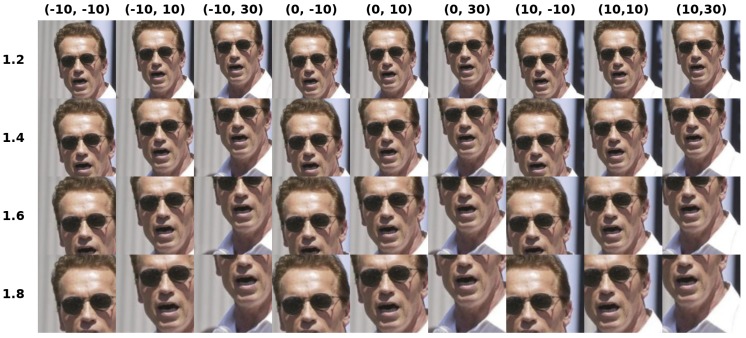
Examples of scale and shift transformations. In vertical we plot changes of the image scales, in horizontal we visualize the shifts.

**Figure 3:**
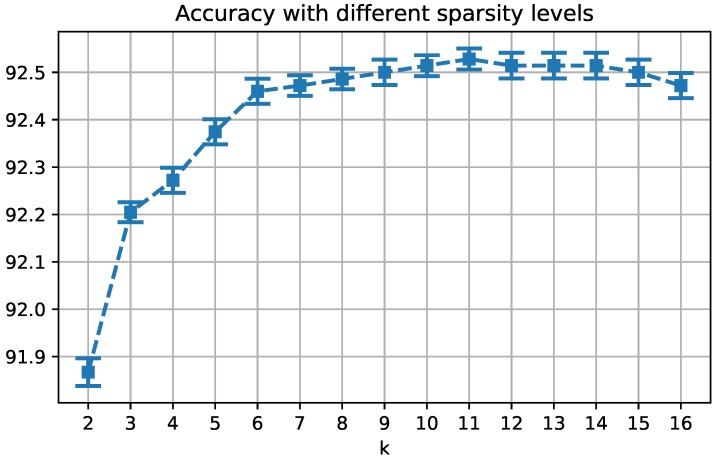
Accuracy of the proposed model on a subset of 100 subjects of the LFW database, varying the value of the parameter *k*.

Given the localized faces, the very first step common to all the experiments concerns with the image augmentation, aiming at generating augmented images of size 224×224, as required by the VGG-face DCNN. In this regard, each image is flipped, resized by a factor in the set {1.2,1.4,1.6,1.8}, shifted horizontally of a quantity within the set {−10,0,+10} pixels, and shifted vertically within the set {−10,+10,+30} pixels. Thus, for each image *I* we attain d=2×4×9=72 augmented images $I_{j}$ and consequently 72 features $\varphi_{j}$ (Figure 2). We notice that, the parameter setting concerning the augmentation step is not critical as long as it allows to catch both local details and holistic information. This is fundamental for dealing with face images presenting partial occlusions or other local variations such as face expression or illumination changes. Indeed, we have tested several configurations either adding or changing the scale (e.g., 2, 2.2, 2.5) or the shifts (e.g., 20, 40), while registering no significant performance differences. This means that such changes add only redundant information. On the contrary, reducing the augmented image set decreases the performance, proving that a certain description richness is useful to the system. The drawbacks of dealing with large dictionaries is the need for managing complex structures of dictionary chunks (like in [9]) and the increase of computational costs. This is solved using the dictionary learning, that compresses all the features in *k* atoms. Experimentally, we set k=6, as a good trade-off between performances and computational costs: as shown in Figure 3, for smaller values we lose effectiveness, while for larger values we do not have a significant gain in performances while the computational costs increase [9].

### 4.1. SSPP with Large Gallery Cardinality

To assess the proposed method performances dealing with galleries with different cardinality, we adopt the LFW dataset. Specifically, we consider the subsets which include no less than 10 or 2 samples per subject respectively (in the following LFW${}_{158}$, and LFW${}_{1680}$), and derive from them the cases with q={100,793}, extracting randomly subsets from LFW${}_{158}$, and LFW${}_{1680}$ respectively. Also the gallery and test construction is generated randomly, and repeated over 10 trials. The average results and the comparisons with the most recent contributions adopting the LFW are reported in Table 1. We observe that our method, besides outperforming all the others, has a slow loss of performances as the gallery cardinality increases. In particular, for the LFW ≤100 we compare our results with the one presented in [20] namely S^3^RC, which is, to the best of our knowledge the state of the art on this particular subset of LFW for the SSPP-FR problem. As can be seen, our method outperforms the other by 1.81%. We believe that this comparison is particularly important given that both methods use the same DCNN features, thus highlighting the robustness of the proposed algorithm. As the cardinality of the gallery increases the gap between our method and the second-best-performing algorithms increases significantly achieving a boost in performance of 63.19% for the LFW${}_{1680}$ subset.

### 4.2. Low-Resolution Test Images

**Figure 4:**
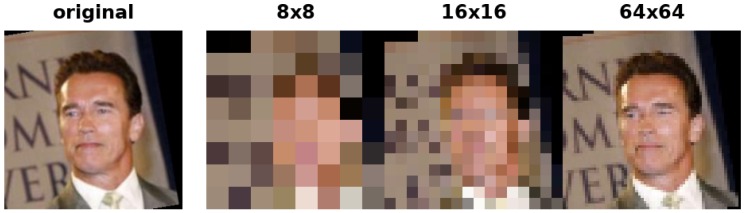
An example of high-resolution images (original), and the corresponding low-resolution ones.

In order to simulate real-world applications where the probe images are captured by surveillance cameras often placed at large distance from the subjects, we setup an experiment where HR images are used for the gallery construction, while LR images are referred to as probe images [6]. Experiments have been conducted adopting the set LFW${}_{158}$, selecting randomly one image for the gallery, and the remaining for test. LR are obtained resizing the images to 64×64 pixels, and then downsampling them to 8×8, and 16×16 pixels (Figure 4).

We conducted two tests, one exploiting the dictionary learned on HR images only, as reported in Section 4.1, and a second, namely SSLD w/LR, where we enlarge the augmented image set of the training set, including the downsampled versions of the reference images. In this last case, the pool of augmented images is d=72+72, that is the set of augmented images obtained from the HR and LR images respectively. The final sub-dictionary dimension *k* remains equal to 6.

In Table 2 we report the obtained results and comparisons. As we can observe, the dictionary learned only on HR images, is suitable to deal with LR images up to a certain level of degradation (i.e., 64 × 64 pixels), losing only 2 percentage points with respect to the result obtained on the HR probe images (cfr. SSLD result in Table 1 for LFW ≤158). On the contrary, when the degradation is stronger, the SSLD system finds hard to determine the correspondences between features extracted from HR in gallery and LR probe images, arriving to a classification drawn by chance for the resolution of (8×8) pixels. In this case, augmenting the reference image descriptions with their LR representations solves part of the ambiguity, producing a great improvement.

The comparison of our method with the one proposed in [6] highlights the effectiveness of the proposed learning procedure. As can be observed (Table 2) the ad-hoc feature augmentation adopted for this experiment (SSLD w/LR) allows to reach a boost in performance of 33.39% and 30.51% if compared with SSLD and [6] respectively, when dealing with face images downsampled to 16 × 16. A similar result is obtained for images downsampled to 8 × 8 pixels; as before the augmentation of the gallery with downsampled images has proven to be useful in classifying LR images obtaining comparable results with the procedure of [6].

### 4.3. Disguised Test Images

**Figure 5:**
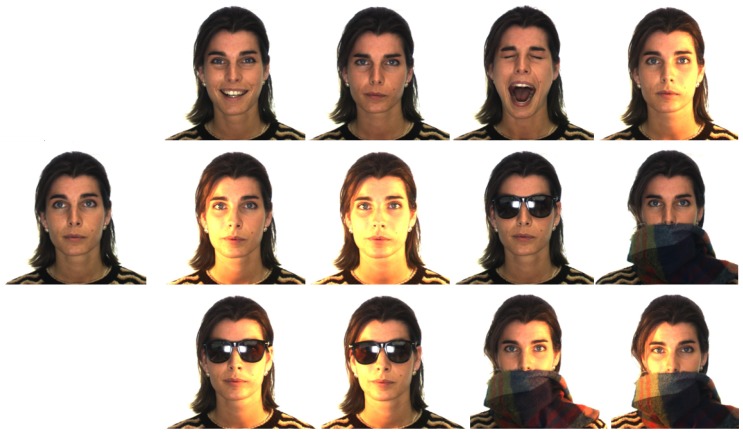
Examples of AR images (session 1). On the left, the neutral image; the others are the test images representing the different categories.

Although the robustness of the SSLD method against possible hurdles is intrinsic in the LFW experiment (Table 1), here we make it explicit by conducting experiments on the distinct categories of the AR database (Figure 5).

In this dataset, each represented individual has been acquired in two sessions, each one composed of 13 different conditions including face expressions (in the following *expr*), illumination changes (in the following *ill*) and partial face occlusions with sunglasses or scarves. In the literature, AR dataset has been adopted also in the SSPP context, even achieving very high performance [18,25,26,44,49]. However this accuracy depends on building models of face variations learned on a subset of subjects showing exactly the same hurdles (same sunglasses, same scarf, same overall conditions) thus not generalizing well. For this reason we take into account only methods without this kind of training.

Experiments have been conducted following the protocols proposed in [23] and [15]: In the first case ([23]) we selected 120 subjects (65 men and 55 women randomly chosen among the 126 available subjects) putting the neutral images of the first session in the gallery, and executing the test on the other images divided per session and category (expr, ill, sunglasses, scarf). In Table 3 results and comparisons with some of the methods outlined in [23] are reported. In the second case, according to [15], we select 100 subjects; for each subject the neutral image of the first session is chosen for gallery and the others (from both session 1 and 2) for test. Differently from the previous setting here we build a category for the occluded images which includes sunglasses and scarf, and a category for occlusions + illumination changes. In Table 4 results and comparisons with some of the methods outlined in [15] are reported.

As can be observed in Table 3, our method is robust with respect to illumination changes, facial expression variations and partial face occlusions, generally outperforming the best method proposed in [23]. This achievement is systematic in the second session, and on average; the second session is in fact harder to classify due to the time which has passed between the acquisition of the two sessions. This is evident in the results summarized in Table 3; we observe that the accuracy heavily drops when classifying face images from the second session in all methods except the one proposed here, this denotes a higher generalization capability and robustness to time variation. Table 4 shows the results of the proposed method in a different setting in which all the occlusions are grouped together and a second group is created for the occluded images with, in addition, changes of lighting. The comparison with the results of [15] shows that our method produces comparable results in terms of accuracy for the expression and occlusion + illumination categories. The LBP + LRA method obtains slightly better results (+2.32%) for the occluded images, while our method largely outperforms the others in the illumination category (+6.69% over the best method). Remarkably, our method is the one that performs better on average, with an overall accuracy that surpasses the best method of [15] by 1.33%.

## 5. Conclusions

This paper presents a method, called SSLD, for solving the SSPP problem coupled with other hurdles which arise from large-scale datasets, large appearance variations (e.g., illumination, facial expression and partial occlusions), and LR probe images. The proposed technique consists in a sparse-driven sub-dictionary learning strategy exploiting the richness of the augmented face image step, the strength of deep features, the simplicity of the MOD technique for sub-dictionary learning, and the effectiveness of the sparse representation via *k*-LiMapS on structured dictionaries. The most time-consuming phase is the dictionary learning, that by the way is worked out only once and offline. On the contrary, the test phase, keeping *k* sufficiently small, can be carried out in real time.

Evaluations have been conducted on the LFW and AR datasets proving that the SSLD method outperforms the state of the art for the SSPP problem, being versatile, data-independent, and scalable. These encouraging results open to further investigations. The first concerns the extension of the SSLD method to deal with even larger galleries (e.g., with 5000 subjects or more). Because of the linear algebra computations, the dictionary learning phase of SSLD would become inefficient dealing with very large galleries, so we plan to study a variant where the reference images are split into several dictionaries to be processed separately and possibly in parallel. Naturally, an integration level is then required to derive the final probe image classification. In addition, other dictionary learning techniques could be conceived, in order to reduce the computational costs and to further improve the system performance. Finally, we observe that the increased performances obtained on LR images when referring to an augmented gallery encoding that hurdle, empirically demonstrates the opportunity to further extend the augmented image set so to be able to capture other form of hurdles that could happen in the test set (e.g., noise, non-homogeneous illumination). This is in the vein of the learning methods presented in Section 2, while having the advantage that we do not rely on a distinct dataset (possibly not representative of the test scenario), being able to model the hurdles on the labeled data themselves.

## Figures and Tables

**Table 1:** Percentages of recognition rate on the LFW dataset, varying the gallery cardinality. For comparison, we report the SSPP state of the art on the LFW. Standard deviation is reported when available. We summarize in a common row results obtaining referring to galleries with slight dimension changes, while precising in brackets the real gallery cardinality. In bold we emphasize the best performance per category.

**LFW ≤ 100 sbj**							
[44]	[45]	[46]	[24]	[26]	[18]	[20]	SSLD
32 (50)	37 (50)	74 (50)	86 (50)	50 (80)	31.39 ± 1.74 (80)	92.57 (100)	**94.38** ± 0.81 (100)
**LFW ≤ 158 sbj**							
[25]		[47]		[48]	[23]	[49]	SSLD
46.3 (120)		27.14 ± 1.0 (150)		30 (158)	37.9 (158)	50 (158)	**92.78** ± 1.2 (158)
**LFW 793 sbj**					**LFW 1680 sbj**		
[24]		SSLD			[16]		SSLD
65.3		**86.43** ± 1.03			21.01		**84.2** ± 0.5

**Table 2:** Experiments on LFW${}_{158}$ with probe images at different level of resolution. In bold we emphasize the best performance per category.

Method	8×8	16×16	64×64
[6]	**12.28**	15.06	-
SSLD	0.74 ± 0.18	12.18 ± 6.89	**90.84** ± 1.13
SSLD w/LR	9.5 ± 0.69	**45.57** ± 1.31	90.62 ± 0.99

**Table 3:** Experiments on AR dataset and comparison with [23]. For each category (Illumination, Expression, Sunglasses, and Scarf) we report the recognition rate (%) for the sessions 1 and 2 (S1, S2), and the average performances (avg.). In bold we highlight the best performances.

Method	Illumination			Expression			Sunglasses			Scarf		
	S1	S2	avg.	S1	S2	avg.	S1	S2	avg.	S1	S2	avg.
SRC	94.70	62.20	78.45	95.30	63.60	79.45	88.10	46.90	67.50	50.60	25.80	38.20
GSRC	96.40	61.10	78.75	94.20	64.20	79.20	84.70	41.40	63.05	46.90	20.60	33.75
LS-MPCRC	98.90	80.0	89.45	**96.90**	80.30	88.60	**97.80**	72.50	85.15	89.40	65.60	77.50
SSLD	**99.66**	**98.33**	**98.99**	95.0	**94.13**	**94.56**	87.0	**83.56**	**85.28**	**97.0**	**90.41**	**93.70**

**Table 4:** Experiments on AR dataset and comparison with [15]. For each category we report the recognition rate on both sessions and the overall accuracy. In bold we highlight the best performances.

Method	Illumination	Expression	Occlusions	Occl + Ill	Overall
Pixel+LRA	72.2	66.0	40.8	19.0	47.8
Gabor+LRA	79.2	93.5	70.3	52.5	72.4
LBP+LRA	92.3	**94.7**	**92.5**	**83.9**	90.1
SSLD	**98.99**	**94.56**	90.18	82.02	**91.43**

## References

[1] Taigman Y., Yang M., Ranzato M., Wolf L. Deepface: Closing the gap to human-level performance in face verification. Proceedings of the 27th IEEE Conference on Computer Vision and Pattern Recognition.

[2] Schroff F., Kalenichenko D., Philbin J. Facenet: A unified embedding for face recognition and clustering. Proceedings of the 28th IEEE Conference on Computer Vision and Pattern Recognition.

[3] Zhao W., Chellappa R., Phillips J., Rosenfeld A. (2003). Face Recognition: A Literature Survey. ACM Comput. Sur..

[4] Lahasan B., Lutfi S.L., San-Segundo R. (2017). A survey on techniques to handle face recognition challenges: Occlusion, single sample per subject and expression. Artif. Intell. Rev..

[5] Ma Z., Ding Y., Li B., Yuan X. (2018). Deep CNNs with Robust LBP Guiding Pooling for Face Recognition. Sensors.

[6] Chu Y., Ahmad T., Bebis G., Zhao L. (2017). Low-resolution Face Recognition with Single Sample Per Person. Signal Process..

[7] Ortiz E.G., Becker B.C. (2014). Face recognition for web-scale datasets. Comput. Vis. Image Understand..

[8] Tan X., Chen S., Zhou Z.H., Zhang F. (2006). Face recognition from a single image per person: A survey. Pattern Recognit..

[9] Bodini M., D’Amelio A., Grossi G., Lanzarotti R., Lin J. (2018). Single Sample Face Recognition by Sparse Recovery of Deep-Learned LDA Features. International Conference on Advanced Concepts for Intelligent Vision Systems.

[10] Parkhi O.M., Vedaldi A., Zisserman A. (2015). Deep face recognition. Proc. Br. Mach. Vis..

[11] Adamo A., Grossi G. A fixed-point iterative schema for error minimization in k-sparse decomposition. Proceedings of the 2011 IEEE International Symposium on Signal Processing and Information Technology (ISSPIT).

[12] Adamo A., Grossi G., Lanzarotti R., Lin J. (2017). Sparse decomposition by iterating Lipschitzian-type mappings. Theor. Comput. Sci..

[13] Engan K., Aase S.O., Husoy J.H. Method of optimal directions for frame design. Proceedings of the 1999 IEEE International Conference on Acoustics, Speech, and Signal.

[14] Grossi G., Lanzarotti R., Lin J. (2017). Orthogonal Procrustes Analysis for Dictionary Learning in Sparse Linear Representation. PLoS ONE.

[15] Deng W., Hu J., Zhou X., Guo J. (2014). Equidistant prototypes embedding for single sample based face recognition with generic learning and incremental learning. Pattern Recognit..

[16] Hu J. (2017). Discriminative transfer learning with sparsity regularization for single-sample face recognition. Image Vis. Comput..

[17] Haghighat M., Abdel-Mottaleb M., Alhalabi W. (2016). Fully automatic face normalization and single sample face recognition in unconstrained environments. Expert Syst. Appl..

[18] Gao S., Zhang Y., Jia K., Lu J., Zhang Y. (2015). Single Sample Face Recognition via Learning Deep Supervised Autoencoders. IEEE Trans. Inf. Forensics Sec..

[19] Deng W., Hu J., Wu Z., Guo J. (2018). From one to many: Pose-Aware Metric Learning for single-sample face recognition. Pattern Recognit..

[20] Gao Y., Ma J., Yuille A.L. (2017). Semi-Supervised Sparse Representation Based Classification for Face Recognition with Insufficient Labeled Samples. IEEE Trans. Image Process..

[21] Yu Y.F., Dai D.Q., Ren C.X., Huang K.K. (2017). Discriminative multi-scale sparse coding for single-sample face recognition with occlusion. Pattern Recognit..

[22] Ji H.K., Sun Q.S., Ji Z.X., Yuan Y.H., Zhang G.Q. (2017). Collaborative probabilistic labels for face recognition from single sample per person. Pattern Recognit..

[23] Liu F., Tang J., Song Y., Bi Y., Yang S. (2016). Local structure based multi-phase collaborative representation for face recognition with single sample per person. Inf. Sci..

[24] Yang M., Wang X., Zeng G., Shen L. (2017). Joint and collaborative representation with local adaptive convolution feature for face recognition with single sample per person. Pattern Recognit..

[25] Ding C., Bao T., Karmoshi S., Zhu M. (2017). Single sample per person face recognition with KPCANet and a weighted voting scheme. Signal Image Video Process..

[26] Gu J., Hu H., Li H. (2018). Local robust sparse representation for face recognition with single sample per person. IEEE/CAA J. Autom. Sin..

[27] Pei T., Zhang L., Wang B., Li F., Zhang Z. (2017). Decision Pyramid Classifier for Face Recognition Under Complex Variations Using Single Sample Per Person. Pattern Recognit..

[28] Wiskott L., Fellous J., Kruger N., von der Malsburg C. (1999). Face recognition by elastic bunch graph matching. Intelligent Biometric Techniques in Fingerprints and Face Recognition.

[29] Perez L., Wang J. (2017). The Effectiveness of Data Augmentation in Image Classification using Deep Learning. arXiv.

[30] Cuculo V., Lanzarotti R., Boccignone G. Using sparse coding for landmark localization in facial expressions. Proceedings of the 2014 5th European Workshop on Visual Information Processing (EUVIP).

[31] Rao C.R. (1948). The Utilization of Multiple Measurements in Problems of Biological Classification. J. R. Stat. Soc..

[32] Fisher R.A. (1936). The use of multiple measurements in taxonomic problems. Ann. Eugenics.

[33] Golub G.H., Van Loan C.F. (2012). Matrix Computations.

[34] Natarajan B.K. (1995). Sparse Approximate Solutions to Linear Systems. SIAM J. Comput..

[35] Grossi G., Lanzarotti R., Lin J. (2015). High-rate compression of ECG signals by an accuracy-driven sparsity model relying on natural basis. Digit. Signal Process..

[36] Adamo A., Grossi G., Lanzarotti R. Sparse representation based classification for face recognition by k-limaps algorithm. Proceedings of the ICISP 2012—International Conference on Image and Signal Processing.

[37] Grossi G., Lanzarotti R., Lin J. (2016). Robust Face Recognition Providing the Identity and Its Reliability Degree Combining Sparse Representation and Multiple Features. Int. J. Pattern Recognit. Artif. Intell..

[38] Adamo A., Grossi G., Lanzarotti R. Local features and sparse representation for face recognition with partial occlusions. Proceedings of the 2013 IEEE International Conference on Image Processing.

[39] Engan K., Aase S.O., Husoy J.H. Designing frames for matching pursuit algorithms. Proceedings of the 1998 IEEE International Conference on Acoustics, Speech and Signal Processing.

[40] Zhang Z., Xu Y., Yang J., Li X., Zhang D. (2015). A Survey of Sparse Representation: Algorithms and Applications. IEEE Access.

[41] Elad M. (2010). Sparse and Redundant Representations.

[42] Huang G.B., Ramesh M., Berg T., Learned-Miller E. (2007). Labeled Faces in the Wild: A Database for Studying Face Recognition in Unconstrained Environments.

[43] Martinez A.M. (1998). The AR Face Database.

[44] Dong X., Wu F., Jing X.Y. (2018). Generic Training Set based Multimanifold Discriminant Learning for Single Sample Face Recognition. KSII Trans. Internet Inf. Syst..

[45] Wang X., Yang M., Shen L., Chang H. Robust local representation for face recognition with single sample per person. Proceedings of the 2015 3rd IAPR Asian Conference on Pattern Recognition (ACPR).

[46] Zeng J., Zhao X., Gan J., Mai C., Zhai Y., Wang F. (2018). Deep Convolutional Neural Network Used in Single Sample per Person Face Recognition. Comput. Intell. Neurosci..

[47] Karaaba M.F., Surinta O., Schomaker L.R.B., Wiering M.A. Robust Face Identification with Small Sample Sizes using Bag of Words and Histogram of Oriented Gradients. Proceedings of the 11th Joint Conference on Computer Vision, Imaging and Computer Graphics Theory and Applications.

[48] Zhu P., Yang M., Zhang L., Lee I.Y., Cremers D., Reid I., Saito H., Yang M.H. (2015). Local Generic Representation for Face Recognition with Single Sample per Person. Computer Vision—ACCV 2014.

[49] Shang K., Huang Z.H., Liu W., Li Z.M. (2018). A single gallery-based face recognition using extended joint sparse representation. Appl. Math. Comput..

